# Integrated Metabolomics and Transcriptome Revealed the Effect of Fermented *Lycium barbarum* Residue Promoting *Ovis aries* Immunity

**DOI:** 10.3389/fimmu.2022.889436

**Published:** 2022-04-08

**Authors:** Yajun Zhang, Yansheng Guo, Yulong Luo, Min Du, Xin Yin, Xiaochun Xu, Guijie Zhang

**Affiliations:** ^1^ Departments of Animal Science, School of Agriculture, Ningxia University, Yinchuan, China; ^2^ School of Food and Wine, Ningxia University, Yinchuan, China; ^3^ Nutrigenomics and Growth Biology Laboratory, Department of Animal Sciences, Washington State University, Pullman, WA, United States; ^4^ State Key Laboratory of Veterinary Biotechnology, Harbin Veterinary Research Institute, The Chinese Academy of Agricultural Sciences, Harbin, China; ^5^ Collaborative Innovation Center for Food Production and Safety, North Minzu University, Yinchuan, China

**Keywords:** *Lycium barbarum* residue, immune response, immunity, immune system, transcriptomic patterns, fermentation, sheep, metabolomics

## Abstract

*Lycium barbarum* residue contains abundant bioactive nutrients which can be used as feed supplement. The fermentation treatment of plant residue can promote the utilization of nutrients, rumen digestion, and the growth and immunity of animals. Based on ultra-performance liquid chromatography-tandem mass spectrometry (UPLC-MS/MS) metabolomics and in-depth transcriptome analysis, the study tested the mechanisms of *Lycium barbarum* residue (RW) and fermented *Lycium barbarum* residue (RFW) on meat quality and immunity of sheep. Fifty-four Tan sheep were randomly divided into control, RFW or RW treatments. Data showed that RFW and RW increased the carcass weight, fat content, ash content and reduced the cooking loss of lamb. RFW performed more significant effects on activating immune-related genes than those of RW. The expression of chemokines and immune-related pathways, such as signaling pathways of interleukin-17 signaling pathway and NOD-like receptor signaling pathway, were elevated in sheep fed RFW. RW increased the diversity in rumen metabolites, especially compositions of lipids, organic acids and organ heterocyclic compounds. RFW affected numerous compounds which are closely correlated with the activation of immune genes. In conclusion, RFW could represent a valuable strategy to improve growth performance and immunity of sheep.

## 1 Introduction

Disease resistance and meat quality are critically important for Tan sheep production. Bioactive metabolites from plants have gained increasing interest as being developed as growth-promoting antibiotics for ruminant animals (1). Various plants have been used as fodder additives for their particular metabolites with antimicrobial, antioxidant, anti-inflammatory and promoting immunity activities (2–5). Thus, developing effective plant-derived fodder additives will improve the production of ruminant animals.

*Lycium barbarum* is one of the important agricultural species, and has been widely used in traditional Chinese medicine. *Lycium barbarum* contains amino acids, polysaccharide (LBP), flavonoids, betaine, vitamins and other active nutrients (6). A wealth of vitamins in *Lycium barbarum* can promote the proliferation of hepatocytes, enhance non-specific immunity, and improve disease resistance and the phagocytosis of phagocytes. In addition, the by-products of *Lycium barbarum* contain a variety of nutrients that could be used as high-quality feed materials for animal. Previous studies have confirmed that the addition of *Lycium barbarum* residue in feed could improve the immunity and growth performance of animals (7). Chemical, physical and biological fermentation could break down the cellulose, hemicellulose, and lignin of plant residues, improving the utilization of nutrients and the efficiency of the animal production. Fermentation technology has been shown to effectively improve the nutrient utilization rate of *Lycium barbarum* and to reduce the feed/gain ratio (8–10). Remarkably, it also can provide readily available nutrients for rumen microorganisms (11, 12), and enhance animal immunity by increasing the levels of immunoglobulin G (IgG), immunoglobulin M (IgM) and interleukin-10 (IL-10) (13). The fermentation treatment of plant residue further promotes the absorption of nutrients and rumen digestion by animals, and has various effects on the growth and immunity of ruminants. Thus, elucidating the mechanisms of *Lycium barbarum* residue and its fermented products on animal immunity and meat quality would contribute to make full use of them and improve animal productivity.

Overall, *Lycium barbarum* residue has the characteristics of green pollution-free, no drug resistance, and can be used as feed ingredients to improve immunity and disease resistance. Although the effect of *Lycium barbarum* residue on the growth and immunity of animals has been investigated, only simple indicators of immunity were revealed, which does not elaborate the mechanism that are sufficient enough to realize the effect of *Lycium barbarum* residue. An integrated investigation of the detailed mechanism of *Lycium barbarum* residue before and after fermentation on sheep immunity remains to be tested.

Recently, with the advancement of multi-omics technology, the changes of animal immune system and meat quality under different conditions can be systematically evaluated, which provides the opportunity for us to deeply understanding the biological process of fermented products *in vivo* (14–17). Based on ultra-performance liquid chromatography-tandem mass spectrometry (UPLC-MS/MS) metabolomics and in-depth transcriptome analysis, the present study investigated the mechanisms of *Lycium barbarum* residue (RW) and fermented *Lycium barbarum* residue (RFW) on meat quality and immunity of sheep. This study aimed to provide the theoretical basis and method for rational utilization of *Lycium barbarum* residue as feed sources.

## 2 Materials and Methods

The experimental protocols were approved by the Institutional Animal Care and Use Committee of Ningxia University (NXUC20200618).

### 2.1 Sample Collection and Preparation

In total, fifty-four ram Tan sheep (120 days, 19.86 ± 0.62 kg) with similar genetic background were selected and randomly divided into 3 experimental groups. Three experimental groups of sheep were feed with basal diet (RCON), basal diet supplemented with *Lycium barbarum* residue (RW) and basal diet supplemented with fermented *Lycium barbarum* residue (RFW) for 70 days. During the feeding trial, sheep were fed twice at 8:00 and 16:00 every day with free access to drinking water. At the end of trial, six biological replicates of sheep in each treatment were selected and slaughtered for further experiment analysis. All sheep were fasted for 16 h before slaughter. Carcass weight was obtained. The rumen was collected for metabolomics and transcriptome analyses, and venous blood was obtained from the jugular vein. The 10 mL venous blood of each sheep was collected. After the blood was fully coagulated, the serum was obtained by centrifugation at 2500 r/min for 15 min and stored at -20°C, then series serum immune indexes were determined, including the contents of immunoglobulin A (IgA), immunoglobulin G (IgG), immunoglobulin M (IgM), interleukin-1β (IL-1β) and interleukin-6 (IL-6).

### 2.2 RNA Extraction

Total RNA was extracted from the rumen of each sample using a total RNA extraction kit (#AM1561, Ambion), following the manufacturer’s instructions. RNA purity was checked using the NanoPhotometer^®^ spectrophotometer (IMPLEN, CA, USA). An Agilent Bioanalyzer 2100 (Agilent Technologies) was used to ensure RNA integrity by determining the RNA integrity number. The sequencing reagent was prepared according to the HiSeq 2500 user guide. Then, clusters were generated.

### 2.3 Transcriptome Sequencing and Assembly

A total amount of 1.5 µg RNA per sample was used as input material for the RNA sample preparations. Sequencing libraries were generated using NEBNext^®^ Ultra™ RNA Library Prep Kit for Illumina^®^ (NEB, USA) following manufacturer’s recommendations and index codes were added to attribute sequences to each sample. The library fragments were purified with AMPure XP system (Beckman Coulter, Beverly, USA) to select cDNA fragments of preferentially 250~300 bp in length. Then 3 µL USER Enzyme (NEB, USA) was used with size-selected, adaptor-ligated cDNA at 37°C for 15 min followed by 5 min at 95°C before PCR. Then PCR was performed with Phusion High-Fidelity DNA polymerase, Universal PCR primers and Index (X) Primer. At last, PCR products were purified (AMPure XP system). The library quality was assessed on the Agilent Bioanalyzer 2100 system.

Raw data (raw reads) of fastq format were firstly processed through in-house perl scripts. In this step, clean data (clean reads) were obtained by removing reads containing adapter, reads containing ploy-N and low quality from raw data. At the same time, Q20, Q30, GC-content and sequence duplication level of the clean data were calculated. All the downstream analyses were based on clean data with high quality. Transcriptome assembly was accomplished based on the left.fq and right.fq using trinity with min kmer cov set to 2 by default and all other parameters set default (18).

### 2.4 Differential Expression Analysis

Gene expression levels were estimated by RSEM for each sample. Clean data were mapped back onto the assembled transcriptome, and read count for each gene was obtained from the mapping results. Differential expression analysis of two conditions/groups was performed using the DESeq R package (1.10.1). DESeq provide statistical routines for determining differential expression in digital gene expression data using a model based on the negative binomial distribution. The resulting *P* values were adjusted using the Benjamini and Hochberg’s approach for controlling the false discovery rate. Genes with FDR < 0.05 and |log_2_FC| >1 found by DESeq were assigned as differentially expressed.

### 2.5 Function Analysis of Differentially Expressed Genes

Genes were classified by GO annotation into three categories: biological process, cellular compartment and molecular function. For each category, a two-tailed Fisher’s exact test was employed to test the enrichment of the differentially expressed proteins against all identified genes. KEGG database was used to identify enriched pathways by a two-tailed Fisher’s exact test to test the enrichment of the differentially expressed genes against all identified genes. The pathways and GO terms with *P* < 0.05 were considered significant. All differentially expressed genes were searched against the STRING database for protein-protein interaction analysis. All interactions with a confidence score ≥ 0.7 (high confidence) were used.

### 2.6 LC-MS/MS Analysis

In total, 1g of rumen from each sample was transferred to an EP tube. After the addition of 400 µL of extract solution (acetonitrile: methanol = 1: 1, containing isotopically-labelled internal standard mixture), the samples were vortexed for 30 s, sonicated for 10 min in ice-water bath, and incubated for 1 h to precipitate proteins. Then the sample was centrifuged at 12,000r/min for 15 min. The supernatant was transferred to a fresh glass vial for analysis. The quality control (QC) sample was prepared by mixing an equal aliquot of the supernatants from all of the samples (19).

LC-MS/MS analyses were performed using an UHPLC system (Vanquish, Thermo Fisher Scientific) with a UPLC BEH Amide column (2.1 mm × 100 mm, 1.7 μm) coupled to Q Exactive HFX mass spectrometer (Orbitrap MS, Thermo). The mobile phase consisted of 25 mmol/L ammonium acetate and 25 ammonia hydroxides in water (pH = 9.75) (A) and acetonitrile (B). The auto-sampler temperature was 4°C, and the injection volume was 3 μL (20). The raw data were converted to the mzXML format using ProteoWizard and processed with an in-house program, which was developed using R and based on XCMS, for peak detection, extraction, alignment, and integration. Then an in-house MS2 database (BiotreeDB) was applied in metabolite annotation. The cutoff for annotation was set at 0.3 (21).

### 2.7 Analysis of Metabolomics Profiles

The analysis of data variation was performed by R packages XCMS software. The Principal Component Analysis (PCA) and pathway enrichment analysis were performed by Metaboanalyst 3.0. PCA was performed on the three-dimensional metabolic data involving the metabolite name, sample name and normalized peak area. The data were further treated through mean centering and unit variance scaling. The PCA plots were generated to interpret cluster separation. And the therapy *P* < 0.05 and fold change (FC) > 2 was used for identification of significantly differential metabolites.

## 3 Results

### 3.1 Dramatic Variations Between RW and RFW in the Transcriptomic Patterns of Sheep

To investigate the effect of RW and RFW on the sheep, rumen with six replicates for each treatment were collected for transcriptome sequencing, and the rumen with basic ration was used as control. In total, 823,571,490 paired-end reads were generated from high-throughput sequencing of all samples, and 44,415,297 clean reads were obtained from 45,753,971 per library in average (**Table 1**). All reads were mapped to the reference genome of sheep (Oar_rambouillet_v1.0), and 95.95% of clean reads were aligned to the genome of sheep in average. Finally, 35,671 genes were obtained for further functional analysis to elucidate the effect of RW and RFW on the transcriptomic pattern of sheep (**Table 1**).

**Table 1:** Characteristics of sheep transcriptome assembly under RFW and RW treatments.

Sample	Raw reads	Clean reads	Clean bases	Total map
**RCON1**	45232800	44092650	6.61G	42788762 (97.04%)
**RCON2**	42219944	40888960	6.13G	39591387 (96.83%)
**RCON3**	43444466	41659558	6.25G	40050506 (96.14%)
**RCON4**	42946476	41714970	6.26G	40189419 (96.34%)
**RCON5**	45723612	44575256	6.69G	43235768 (96.99%)
**RCON6**	45899104	44166626	6.62G	42598564 (96.45%)
**RW1**	45946156	44674048	6.7G	42923559 (96.08%)
**RW2**	53574850	51574902	7.74G	49474581 (95.93%)
**RW3**	44084436	42681690	6.4G	41138358 (96.38%)
**RW4**	47272822	45844162	6.88G	44022901 (96.03%)
**RW5**	47793996	46327334	6.95G	44360148 (95.75%)
**RW6**	46289582	45130724	6.77G	43245909 (95.82%)
**RFW1**	46547690	45206554	6.78G	42911105 (94.92%)
**RFW2**	44450046	43092694	6.46G	40130860 (93.13%)
**RFW3**	46325768	45126570	6.77G	43229285 (95.8%)
**RFW4**	46189858	44887138	6.73G	43013512 (95.83%)
**RFW5**	46195812	44935596	6.74G	43096286 (95.91%)
**RFW6**	43434072	42895926	6.43G	41031677 (95.65%)

**Figure 1:**
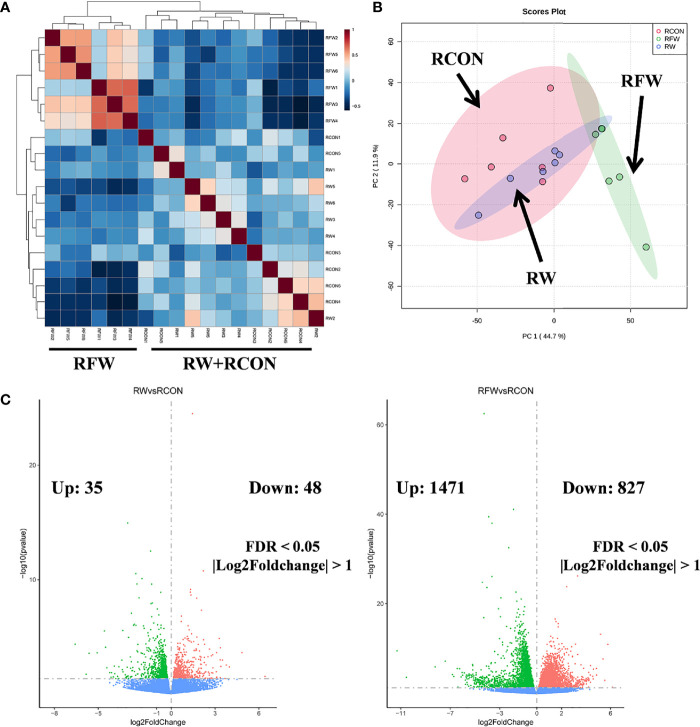
Landscape of the transcriptomic patterns of sheep after RFW and RW treatments. **(A)** Correlation analysis on all samples showing two distinct clades: samples of RFW and RW+RCON. The correlation value between samples were represented by the color of each cell. **(B)** Principal component analysis (PCA) on the metabolic profiles of sheep after RFW and RW treatments. Three regions within 95% confidence intervals were formed in the PCA plots, including RCON, RW and RFW. **(C)** Volcano plots showed the differentially expression genes in RW *vs.* RCON (left) and RFW *vs.* RCON (right) pairwise comparisons. The upregulated and downregulated genes were shown in pink and green, respectively, whereas blue dots represented the genes without significance in each comparison.

Subsequently, based on the transcriptome profiles of the three experimental treatments of RW, RCON and RFW, two algorithms, correlation analysis and PCA, were further used to study the overall impact of RW and RFW treatments on the transcriptome profiles of sheep (**Figure 1**). The correlation analysis displayed an obvious cluster containing all samples from RFW treatment, whereas samples from other two treatments (RW and RCON) irregularly clustered into another cluster (**Figure 1A**). This suggests that RFW treatment caused more significant variations in transcriptomic patterns of sheep than those of RW and RCON treatment. Further PCA analysis reached a consensus with the correlation analysis that all RW and RCON samples distributed into one region, whereas RFW samples separated with both groups and formed another region (**Figure 1B**). The top two components of PCA analysis explained 44.7% and 11.9% of the variances caused by RFW and RW treatments, represented by principal component PC1 and PC2 (**Figure 1B**). PCA analysis supported that RFW caused more transcriptomic variations of sheep than those of RW treatment. Then, we set threshold value of FDR < 0.05 and |log_2_FC| > 1 to identified the differentially expression genes (DEGs) in two pairwise comparisons (**Figure 1C**). Compared with RCON, 83 genes were altered to differentially express in RW treatments, with 35 upregulated genes and 48 downregulated genes (**Figure 1C**). By contrast, RFW dramatically induced variations in gene expression of 2,298 genes, with 1,471 upregulated genes and 827 downregulated genes (**Figure 1C**). Thus, these DEGs from both pairwise comparisons were used for further function analysis to elucidate the detailed effects of RW and RFW treatments on sheep.

#### 3.1.1 RW Altered Transcription Patterns of Metabolisms Relevant to Amino Acids and Fatty Acids

**Figure 2:**
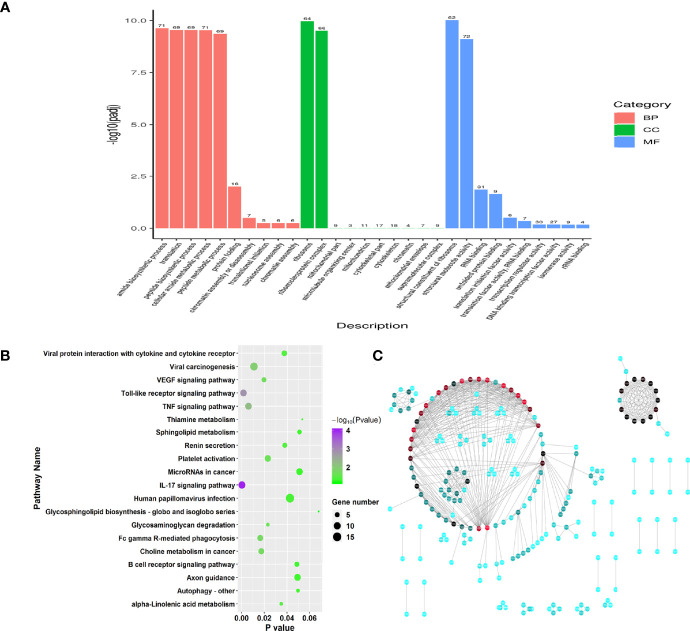
Function enrichment analysis on the significant differential genes in RW *vs.* RCON comparison. **(A)** Gene ontology enrichment analysis on all differential genes in RW *vs.* RCON comparison. Three main kinds of terms relevant to biological process, cellular component and molecular function were shown in pink, green and blue, respectively. The vertical coordinate represented the *P* value of each GO term. **(B)** Dot plots showing the results of KEGG pathway enrichment analysis on upregulated genes upon RW treatment. The dot size represented the gene number involved in each pathway, whereas the dot color represented the *P* value of each pathway. The abscissa represented the *P* value. **(C)** Protein-protein interaction network of RW-associated genes. The dot color represented the importance of each gene in this topological network.

To investigate the function of genes of RW treatments, we performed Gene Ontology (GO) enrichment analysis on the 83 DEGs. Functional enrichment results showed that these DEGs (FDR < 0.05) were involved in 805 GO categories, with 35 significant GO terms among three main groups, including biological process, molecular function and cellular component (**Supplementary Table 1**, *P* < 0.05). Two significantly enriched terms relevant to cellular component were ribosome and ribonucleoprotein complex (**Figure 2A**). Among molecular function categorizes, 13 terms were significantly enriched in the RW *vs.* RCON comparison, including structural constituent of ribosome, structural molecule activity, RNA binding, unfolded protein binding, translation initiation factor activity, translation factor activity, RNA binding, transcription regulator activity, DNA binding transcription factor activity, isomerase activity, rRNA binding, oxidoreductase activity, chemokine activity and chemokine receptor binding (**Figure 2A**). We noted that RW mainly altered genes with chemokine and transcription activity, demonstrating that RW might affect the chemokine production of sheep. For biological processes, 20 categories were significantly identified, especially genes with protein modification and transcription activity, such as amide biosynthetic process, translation, peptide biosynthetic process, cellular amide metabolic process, peptide metabolic process, protein folding, chromatin assembly or disassembly, translational initiation, nucleosome assembly, chromatin assembly, protein-DNA complex assembly and regulation of hydrolase activity (**Figure 2A**).

Subsequently, pathway enrichment analysis was performed on the upregulated and downregulated genes in RW *vs.* RCON comparison based KEGG database. 35 upregulated genes were enriched in 257 pathways, with 16 significant pathways (**Figure 2B**, *P* < 0.05). We noted various immune-related pathways were significantly activated upon RW treatment, such as IL-17 signaling pathway, Toll-like receptor signaling pathway, TNF signaling pathway, autophagy and phagocytosis, as well as the expression of cytokines (**Figure 2B**). In addition, RW also altered the gene expression relevant to metabolisms, such as glycosaminoglycan degradation and alpha-linolenic acid metabolism (**Figure 2B**). Further pathway analysis on 48 downregulated genes showed that RW-suppressed genes mainly functioned in pathways relevant to metabolisms. Various pathways relevant to amino acid and fatty acid metabolisms, such as valine, leucine and isoleucine degradation, butanoate metabolism, sulfur metabolism, fatty acid degradation, propanoate metabolism, tryptophan metabolism and fatty acid elongation, thiamine metabolism, were significantly enriched (**Supplementary Figure 1A**). Meanwhile, these genes involved in a duplicated interaction network *in vivo*, suggesting that RW altered transcriptional patterns in sheep on a global scale, leading to activation and metabolic redirection of immune-related genes associated with fatty acids and amino acids in sheep (**Figure 2C**).

#### 3.1.2 RFW Triggered Expression of Genes Involved in Sheep Immune System

**Figure 3:**
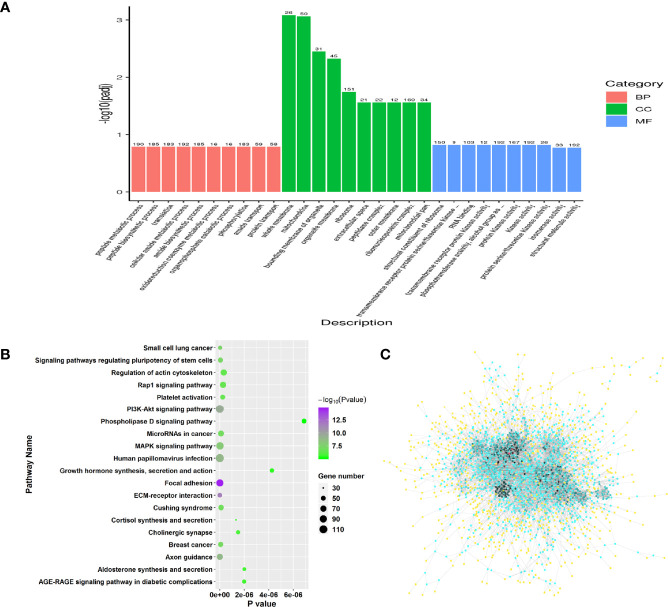
Genes triggered by RFW treatments mainly involved in the immune system of sheep. **(A)** GO enrichment analysis on all DEGs from RFW *vs.* RCON comparison. Biological process, cellular component and molecular function terms were shown in pink, green and blue, respectively. The vertical coordinate represented the *P* value of each GO terms. **(B)** Dot plots showing the results of KEGG pathway enrichment analysis on upregulated genes upon RFW treatment. The dot size and color represented the gene number and *P* value of each pathway, respectively. The abscissa represented the *P* value. **(C)** Protein-protein interaction network of RFW-associated genes. The dot color represented the importance of each gene in this topological network.

Similarly, GO and pathway enrichment analysis were also performed in RFW *vs.* RCON comparison to investigate the effect of RFW on transcriptomic variations of the sheep. GO enrichment analysis on 2,298 DEGs showed that RFW mainly altered 157 GO terms among biological process, molecular function and cellular component (**Supplementary Table 2**, *P* < 0.05). For biological process, various terms relevant to transcription and metabolisms were affected by RFW treatment, including peptide metabolic process, translation, amide biosynthetic process, phosphorylation, nucleoside phosphate catabolic process, protein localization, intracellular transport, pyridine nucleotide biosynthetic process, ATP metabolic process, cation homeostasis, purine ribonucleotide biosynthetic process, cell differentiation, coenzyme biosynthetic process and lipid catabolic process (**Figure 3A**). The various related cellular component terms were significantly enriched, including whole membrane, mitochondrion, organelle membrane, ribosome, extracellular space, peptidase complex, mitochondrial outer membrane, envelope, cytoplasmic vesicle part and golgi-associated vesicles (**Figure 3A**). Similarly, RFW also affected various genes with transcription function. Numerous molecular function related terms were significantly identified in RFW treatment, such as structural constituent of ribosome, transmembrane receptor protein serine/threonine kinase activity, RNA binding, protein kinase activity, 5’-3’ RNA polymerase activity, RNA polymerase activity and ferric iron binding (**Figure 3A**).

Remarkably, the pathway analysis of 1,471 upregulated genes were mainly involved in various immune-related pathways, such as, IL-17 signaling pathway, NOD-like receptor signaling pathway and Chemokine signaling pathway (**Figure 3B**). Various studies have well documented these pathways in animal immune system (22–25). The 1,471 upregulated genes also involved in a complex interaction network (**Figure 3C**), demonstrating the effect of RFW on the sheep immune system were comprehensive. Remarkably, RFW activated more immune-related pathways and the activation degree was more significant than RW treatment (**Figure 3B**). In parallel, among the 827 downregulated genes, pathway analysis showed that some metabolism-related pathways were significantly inhibited *in vivo*, while immune-related pathways have not identified significantly in RFW treatment (**Supplementary Figure 1B**). Furthermore, we determined the levels of immune indexes under RW and RFW treatments. The results showed that both RW and RFW treatments could increase the IgG, IgA, IgM and GLB levels. The effect of RFW was more significant in increasing immune-related factors, with 242 μg/mL of IgG, 135 μg/mL of IgA, 105 μg/mL of IgM and 34.9 μg/mL of GLB (**Table 2**). It supported that RFW could trigger the expression of more immune-related genes in sheep.

**Table 2:** Influences of RFW and RW on the immune indicators of sheep.

Name	RCON	RW	RFW	SEM	*P-*value
IgG μg/mL	170.56^a^	200.03^b^	242.28^b^	39.61	0.04
IgA μg/mL	102.48	110.03	135.49	23.55	0.26
IgM μg/mL	53.54^a^	76.72^ab^	105.04^b^	20.67	0.01
GLB g/L	29.58^a^	31.00^b^	34.95^b^	2.48	0.04

^a,b^Different letters within a row means values with significantly different (P < 0.05).

#### 3.1.3 Effects of RFW and RW on Acting Immune-Related Genes

**Figure 4:**
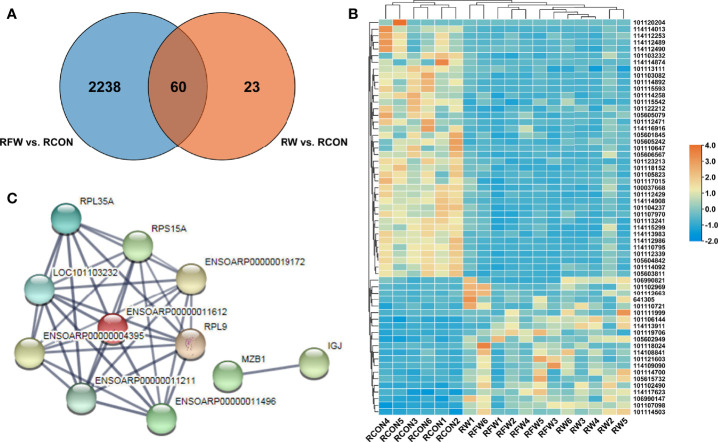
Immune-related genes were shared in RFW *vs.* RCON and RW *vs.* RCON comparisons. **(A)** Venn diagram showed that genes shared in RFW *vs.* RCON and RW *vs.* RCON pairwise comparisons. The DEGs from RFW *vs.* RCON and RW *vs.* RCON were represented by blue and orange circles, respectively. **(B)** Heatmap displayed the expression pattern of 60 DEGs shared in RFW *vs.* RCON and RW *vs.* RCON pairwise comparisons. The up- and down-regulated genes were shown in red and blue, respectively. The gene expression levels were represented by the normalized FPKM values of gene in each sample. **(C)** The protein-protein interaction network of the genes shared in both comparisons.

To investigate whether RFW and RW could trigger common immune-related response, we identified the 60 DEGs that were common in RFW *vs.* RCON and RW *vs.* RCON comparisons. As shown in **Figure 4A**, RFW and RW specifically affected 2,238 and 23 genes, respectively. Notably, RFW and RW had similar effects on these genes, which were both induced or suppressed by RFW or RW in sheep (**Figure 4B**). Protein-protein interaction analysis showed that these genes were involved in a simple network with only 11 proteins that could interact with others (**Figure 4C**). Further pathway enrichment analysis of these genes showed that these genes were mainly involved in ribosome, and no obvious immune or metabolism-related pathways were identified in these 60 genes (**Table 3**). These results suggested that RFW could specifically trigger the immune response and promote the immunity of sheep. Although RW could also trigger immune-related genes *in vivo*, its effects were not as obvious as RFW. RW mainly altered the metabolic flux of sheep, which may cause differences in meat quality.

**Table 3:** Pathway enrichment results of genes involved in the protein-protein interaction network.

Pathway	Gene count	FDR	Matching proteins in your network (labels)
**Ribosome**	5	0.00036	RPL9, ENSOARP00000004395, RPS15A, LOC101103232, RPL35A

### 3.2 Dramatic Variations Between RW and RFW in the Metabolic Patterns of Sheep

**Figure 5:**
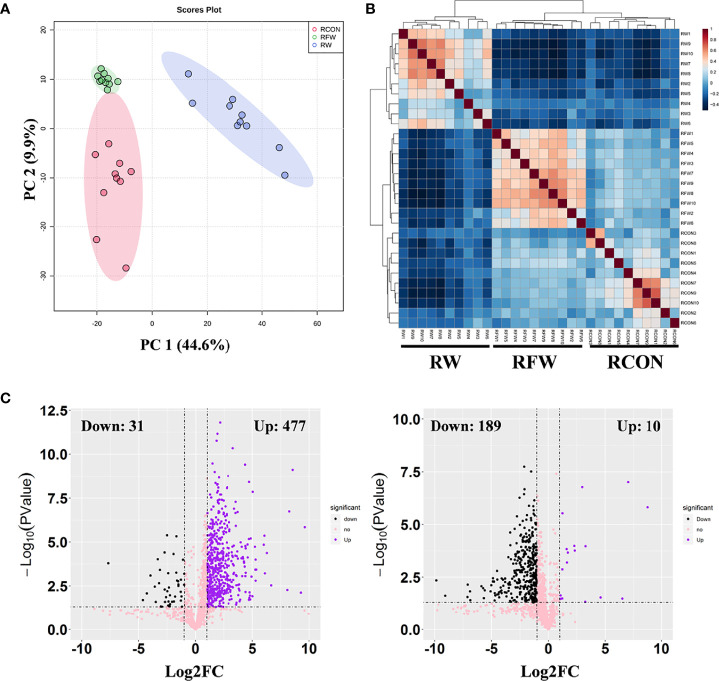
Overview of metabolic profiles of rumen after RFW and RW treatments. **(A)** Principal component analysis on the metabolic profiles from three experiment treatments. The PCA plots showed the separation of three treatments and stability of replicates from same treatment. Each dot represented a sample. Three ellipses were the 95% confidence intervals formed in the PCA plots, representing RCON, RW and RFW treatments. PC1 for metabolomics explains 44.6% of variance and PC2 explains 9.9% of variance using integral metabolomics data. **(B)** Heatmap showing the square of Pearson correlation coefficient between samples. The correlation analysis showed tree clades in the heatmap, representing biological replicates from three experiment treatments. **(C)** Volcano plots of differential metabolites in RFW *vs.* RCON and RW *vs.* RCON pairwise comparisons. Each plot represented a metabolite identified in metabolomics profiles. The increased and decreased metabolites were shown in purple and black dots. The metabolites without significance were shown in pink.

Presently, we found that RFW and RW treatment could increase the carcass weight, fat content and ash content (**Table 4**). The increase of these three indicators was higher in RFW than in RW treatment. Meanwhile, RFW and RW treatments could reduce the cooking loss of sheep (**Table 4**). Thus, we concluded that RFW and RW could affect the meat quality. Numerous indicators of meat quality were closely associated with the changes in metabolisms *in vivo*. Considering the influences of RFW and RW on metabolisms of sheep at transcription levels, we performed metabolomics analysis on the rumen in RFW and RW treatment after 70 days (**Figure 5**). Ten replicates of each treatment were collected for metabolomics analysis based on high resolution LC-MS platform. The rumen with basic ration was set as control (RCON). Totally, 329 and 1,034 metabolites were identified in negative and positive modes, respectively (**Figure 5**). As shown in **Figure 5A**, PCA analysis was conducted on the metabolic profiles among three treatments. The results indicated that the metabolic patterns of three treatments were significantly different, and the three treatments were distributed in different regions. The differences may be concentrated in a few highly affected metabolites or may exist in all metabolic patterns. Remarkably, the region of RFW-treated samples was close to the RCON region, indicating similar metabolic patterns between the two groups (**Figure 5A**). However, RW-treated samples distinctly separated with RCON and RFW, which was consistent with the transcriptomic results that RW altered more genes involved in metabolic pathways than those of RFW and RCON treatments (**Figure 5A**). The top two main components could explain 54.5% of the RFW/RW-associated variations in the data (**Figure 5A**). Further unsupervised hierarchical clustering analysis was performed to identify the variances of metabolic patterns among both RFW *vs.* RCON and RW *vs.* RCON pairwise comparisons (**Figure 5B**). Data showed that all samples under the same treatments were closely clustered on one branch, and the comparison difference between RW *vs.* RCON was more significant than that between RFW *vs.* RCON (**Figure 5B**). These data were in good agreement with the PCA results.

**Table 4:** Effects of RFW and RW treatments on slaughter performance of sheep.

Term	RCON	RW	RFW	SEM	*P-*values
**Carcass weight (kg)**	15.32^a^	16.83^b^	16.88^b^	0.53	0.04
**Pure meat percentage (%)**	63.54	61.81	63.04	2.34	0.87
**Cooking lost (%)**	46.91^a^	44.39^b^	44.05^b^	0.48	0.02
**Water content (%)**	74.17^a^	75.37^b^	75.05^b^	0.48	0.04
**Fat (%)**	14.85^a^	17.17^b^	18.47^b^	1.76	0.02
**Ash content (%)**	4.01^a^	4.57^b^	4.61^b^	0.07	0.03

^a,b^Different letters within a row means values with significantly different (P < 0.05).

Subsequently, we set threshold (|log_2_FC| > 1, *P*-value < 0.05, and VIP > 1) to identified differential metabolites in both comparisons (**Figure 5C**). The VIP values of each metabolite were calculated from the PLS-DA model. A total of 508 metabolites changed significantly between the RW and RCON treatment, with 31 downregulated metabolites and 477 upregulated metabolites (**Figure 5C**). However, RFW only significantly altered the levels of 199 metabolites, with 189 metabolites decreased and 10 metabolites increased (**Figure 5C**). Therefore, we concluded that the metabolic patterns of sheep in RFW and RW treatments were significantly different, and RW had a greater impact on the metabolic patterns of sheep.

#### 3.2.1 Compositions of Fatty Acids and Amino Acids in Rumen

**Figure 6:**
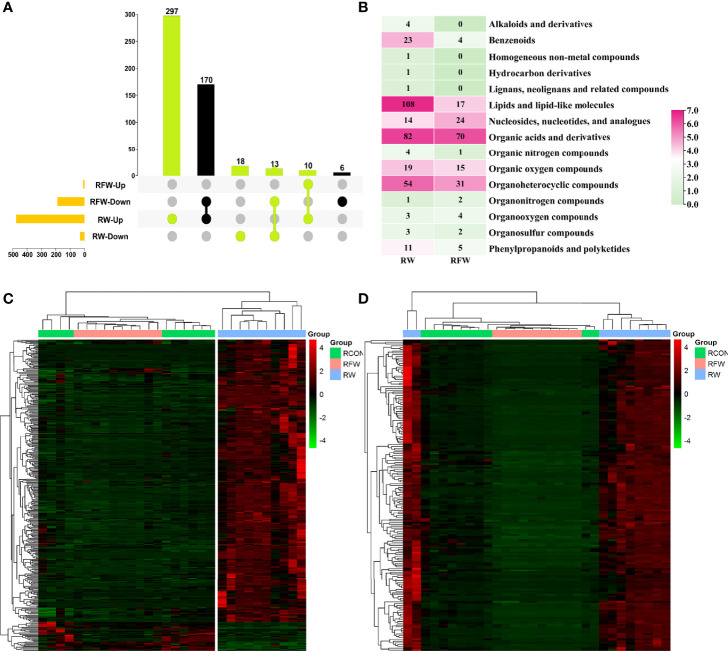
Identification of candidate RFW- and RW-associated metabolites. **(A)** Upset diagram showed the specific metabolites identified in RFW *vs.* RCON and RW *vs.* RCON pairwise comparisons. RFW- and RW-associated metabolites were shown in black and light green, respectively. **(B)** Classifications of metabolites closely correlated with RFW and RW treatments. The color represented the metabolite number in each classification, and the number were shown in each cell. **(C, D)** Heatmap displayed the relative level of RW **(C)** and RFW -associated **(D)** metabolites. The metabolites with high levels were shown in red, whereas the metabolites with low levels were shown in green. The normalized peak area of each metabolite was used to represent the levels.

In this study, we identified 338 and 176 metabolites which were closely associated with RW and RFW treatment, respectively (**Figure 6A**). Among 338 RW-related metabolites, 297 compounds were specifically induced by RW treatment that they increased to high levels (**Figures 6A, C**). 18 metabolites were specifically decreased in RW treatment (**Figures 6A, C**). 10 metabolites were commonly upregulated in RW and RFW treatment, while 13 metabolites were commonly downregulated in RW and RFW treatment (**Figures 6A, C**). Additionally, we identified 176 metabolites in the RFW treatment (**Figure 6A**). Among these 176 metabolites, 6 compounds were specifically decreased in RFW treatment, whereas 170 metabolites were downregulated by RFW treatment and upregulated by RW treatment (**Figures 6A, D**). We recognized these 176 metabolites as the candidate metabolites which contributed to activation of immune responses by RFW treatment. The 338 metabolites were the candidate compounds which explained the influences of RW on the metabolic pattern of sheep.

Subsequently, we analyzed the categorizes of these two sets of metabolites (**Figure 6B**). The results showed that among 338 RW-related metabolites, lipid, organic acid and organ heterocyclic compound-related metabolites were the main components, of which 108 and 82 compounds belonged to both categorizes of metabolites (**Figure 6B**). Thus, it suggested that RW could alter the composition of lipid and organic acid to affect the meat quality of sheep. It has been reported that alterations in lipid, amino acid and organic acid-related metabolites could affect the meat quality of various ruminant animal (26, 27). Additionally, benzenoids, organic oxygen compounds and nucleotides also accounted for a certain percentage (**Figure 6B**). By contrast, RFW had no significant effect on lipid composition in sheep, only on 17 lipid levels (**Figure 6B**). Similarly, RFW also significantly affected the levels of 70 organic acid and 31 organ heterocyclic compound-related metabolites (**Figure 6B**). However, the overall metabolic changes caused by RFW were significantly less than those of RW treatment.

#### 3.2.2 The Immune System and Metabolism Network

**Figure 7:**
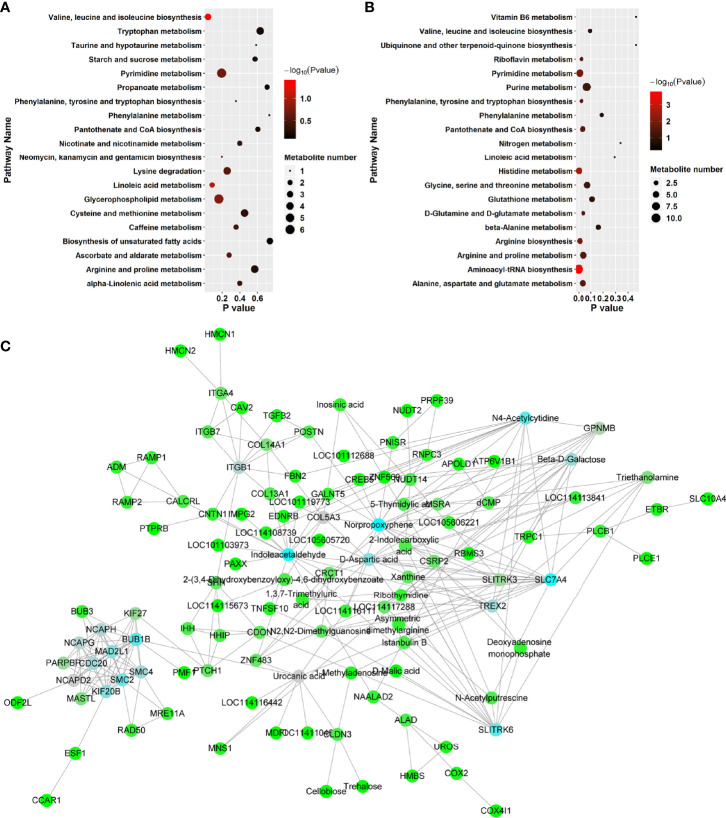
RFW and RW treatments exhibited different effects on metabolic compositions and immune system of sheep. **(A, B)** Top 20 of enriched pathways of RW- **(A)** and RFW-associated **(B)** metabolites; The size of circle represented the number of metabolites in the related pathways. And the significance was shown in different color (high: black, low: red, *P* < 0.05). **(C)** Coexpression network of RFW-associated metabolites and RFW-triggered immune genes. The hub metabolites and genes in the topological network were shown in blue.

KEGG pathway enrichment analysis was performed to investigate the detailed pathways involved in these RFW and RW-related metabolites *in vivo* (**Figure 7** and **Table 5**). The results showed that RW-related metabolites were involved in 43 biosynthetic pathways, in which the biosynthesis of valine, leucine and isoleucine and the metabolism of linoleic acid were all highly enriched (**Figure 7A**). The biosynthesis of valine, leucine and isoleucine was the most significant pathway, *P* < 0.05 (**Figure 7A**), indicating that RW mainly altered the biosynthesis of valine, leucine and isoleucine in sheep, resulting in changes in meat quality. Remarkably, the top 10 related pathways mainly involved in amino acid and fatty acid metabolism, including valine, leucine and isoleucine biosynthesis, linoleic acid metabolism, glycerophospholipid metabolism, pyrimidine metabolism, lysine degradation, ascorbate and aldarate metabolism, phenylalanine, tyrosine and tryptophan biosynthesis, caffeine metabolism, alpha-linolenic acid metabolism, cysteine and methionine metabolism and arginine and proline metabolism (**Figure 7A**). Subsequently, pathway analysis results also displayed overrepresentation of pathways involved in primary biosynthesis based on RFW-related metabolites (**Figure 7B**). Series amino acid-related pathways were significantly affected by RFW treatments, including histidine metabolism, arginine biosynthesis, phenylalanine, tyrosine and tryptophan biosynthesis, alanine, aspartate and glutamate metabolism, arginine and proline metabolism and D-Glutamine and D-glutamate metabolism (**Figure 7B**). It suggested that RFW also affected the meat quality by redirecting amino acid composition and may trigger immune-related genes through this strategy. Additionally, glycine, serine and threonine metabolism, valine, leucine and isoleucine biosynthesis, glutathione metabolism, beta-alanine metabolism and phenylalanine metabolism were also altered (**Figure 7B**). However, we did not identify fatty acid-related pathways in RFW-related metabolites (**Figure 7B**), indicating that RFW did not affected the meat quality by changing fatty acid composition *in vivo*. The aminoacyl-tRNA biosynthesis pathway was the most significantly affected pathway in RFW treatment, *P* < 0.01 (**Figure 7B**). Various researches suggested that aminoacyl-tRNA biosynthesis could influenced the immune responses (28–30). Thus, we analyzed the correlation between RFW-related metabolites and RFW-triggered immune genes (**Figure 7C**). The coexpression network analysis revealed that the metabolites were closely associated with immune-related genes (**Figure 7C**), suggesting that RFW could reorganize the transcriptome to influence metabolic composition and trigger immune-related responses. Furthermore, we identified 71 hub metabolites such as 2-(3,4-Dihydroxybenzoyloxy)-4,6-dihydroxybenzoate, triethanolamine, indoleacetaldehyde, urocanic acid, istanbulin B, 2-Indolecarboxylic acid, norpropoxyphene and Beta-D-galactose, which may be related to the activation of immune-related genes (**Supplementary Table 3** and **Figure 7C**). These metabolites mainly derived from purine metabolism and pyrimidine metabolism, and some metabolites were derived from amino acid metabolisms (**Supplementary Table 3** and **Figure 4**). In conclusion, both RFW and RW treatments affected amino acid composition, and RW could also affect fatty acid composition. RFW may change the serial metabolism of purines and pyrimidines in sheep, thereby exerting its immune system-promoting effect.

**Table 5:** Pathway enrichment results on hub metabolites involved in the topological network.

Pathway	Hits	*P*-value	Impact
Purine metabolism	3	0.046	0.156
Pyrimidine metabolism	2	0.080	0.086
Caffeine metabolism	1	0.141	0.000
Histidine metabolism	1	0.184	0.123
Starch and sucrose metabolism	1	0.204	0.000
Alanine, aspartate and glutamate metabolism	1	0.300	0.000
Arginine and proline metabolism	1	0.385	0.023
Tryptophan metabolism	1	0.408	0.013

## 4 Discussion

### 4.1 The Effects of RFW on IgA and IgG of Sheep

Traditional technology for the usage of plant-derived feed resources could not meet the development of animal feeding industry. However, the advanced fermentation technology contributed the usage of plant-derived fodder source, improving resource utilization rate and saving feed costs. Although this study did not reveal the mechanism of how fermented *Lycium barbarum* residue improves meat productivity of sheep without changing rumen metabolism, it provided plenty of evidences to decipher the detailed metabolic changes of rumen caused by fermented and non-fermented *Lycium barbarum* residue. Importantly, the addition of RFW can effectively improve the overall immunity of sheep, which is conducive to improving the resistance of sheep to a variety of pathogenic microbial and diseases in production sheep. Fermented feed refers to artificially controlled feed with microorganisms and enzymes as starter agents. The nutrients and anti-nutritional factors of feed raw materials could be decomposed or transformed into biological feed containing microbial protein and bioactive peptide amino acids, which were easy for animals to digest and absorb without toxic effects. *Lycium barbarum* residue is rich in nutrients and beneficial metabolites, which are more conducive to animal rumen digestion and absorption after fermentation. It could improve animal growth performance, daily gain, composition of rumen microflora and reduce the ratio of feed consumption to gain (8–10). Importantly, the use of *Lycium barbarum* residue and its fermented products can effectively improve animal immunity. The levels of immunoglobulin in animal blood directly reflect the immune activity of animals. Previous studies found that *Lycium barbarum* residue can increase the levels of IgA and IgG in serum of Tibetan sheep, thereby improving immunity (31, 32). Similarly, our results also showed that fermented *Lycium barbarum* could promote the levels of IgA and IgG of sheep.

### 4.2 The Effects of RFW on Acting Immune-Related Genes

Various studies have shown that the increases in immunoglobulin *in vivo* is mainly caused by the high levels of *Lycium barbarum* polysaccharide (1, 31). Our study showed that RFW-induced multiple carbohydrates are involved in the network of immune-related genes and metabolites, such as cellobiose, Beta-D-galactose and trehalose. Researcher proved that *Lycium barbarum*-related polysaccharide protein complex (LBP3P) can promote the expression of interleukins and TNF-α at mRNA and protein levels, regulate the immune response of animal, and improve immune performance (32). Additionally, our results showed that RFW triggered various immune-related genes involved in PI3K-Akt signaling pathway, Rap1 signaling pathway, Hedgehog signaling pathway, Wnt signaling pathway, Hippo signaling pathway, IL-17 signaling pathway, and Chemokine signaling pathway. As an important part of cellular immunity, cytokines are divided into anti-inflammatory cytokines and pro-inflammatory cytokines (IL-1β, IL-6 and tumor necrosis factor α, TNF-α), which have the functions of regulating immunity, hematopoiesis and anti-inflammatory effect (33). Immunoglobulin is an important part of humoral immunity and plays an important role in the body’s immune defense. Similarly, researchers showed that fermented wheat bran increased the levels of IgG, IgM and interleukin-10, but did not affect the levels of IL-1β, IL-6 and TNF-α, indicating that fermented wheat bran could stimulate the secretion of immunoglobulin and anti-inflammatory cytokines (13). The researchers also confirmed that fermented feed can significantly increase the serum IgG and IgM levels of sheep, as well as the level of IL-6. The above results showed that fermented feed can improve the immune level of sheep to a certain extent, which may be because fermentation promotes the absorption of nutrients, thereby improving the immunity of animals. Thus, we proposed that RFW has specific metabolic components and is more advantageous than RW in improving immunity in sheep.

### 4.3 The Effects of RFW and RW on Ruminal Metabolic Patterns

We compared the effects of RW and RFW on ruminal metabolic patterns. RW induced more striking variations in metabolic patterns than that of RFW treatment. We assumed that this variation is mainly caused by the degradation of *Lycium barbarum* residue caused by fermentation. Indeed, fermentation could enormously change the physical and chemical properties of plant residues, leading more conducive absorption of nutrients which have been degraded into macromolecules with more comfortable solubility and sizes (34). *Bacillus siamensis* involved fermentation could make the soybean residue loosened and poriferous, leading increases of crude protein and small peptide and decreases of antinutritional factor globulins in fermented soybean residue, which improved the digestibility of sheep (35). Meanwhile, the crude fat content in fermented corn and soybean meal mixture were lower than that in unfermented feed, leading increases of ash, calcium and total phosphorus contents of pig meats during lactation (36). Similarly, RFW and RW also caused the increases of carcass weight, fat and ash contents. Considering the degradation of 80% anti-nutritional factors in soybean residue caused by fermentation (36), thus we proposed that RFW also promoted the degradation of anti-nutritional factors and macromolecular fat, which provided a shortcut for nutrients uptake and economized energy expense in comparison to RW treatment. Indeed, non-fermented *Lycium barbarum* residue will also be fermented in rumen of ruminants for further absorption of nutrients. Due to the large number and variety of microorganisms in the rumen, it is a natural anaerobic fermenter and is also the main place for material degradation and nutrient absorption (37). Meanwhile, rumen also provides a suitable environment for the development of microbes, in return these microbials further functioned *in vivo* to affect the host immunity and homeostasis (38). Considering the conducive nutrient absorption of RFW, the microbials could deploy the nutrient more fully in rumen, resulting in the promotion of sheep immunity and meat performance. However, RW treatment also compelled rumen to burden the task for disintegration of organic macromolecules, which could be dealt by fermentation. Integrated metabolomics and transcriptome showed the activation of series nutrient and energy-related pathway in RW treatment, supporting our hypothesis that rumen need to deploy more resources to participate in the assimilation process and nutrient absorption. Overall, RWF treatment could improve the physicochemical properties and structure of *Lycium barbarum* residue and optimize its nutrient composition, contributing to the utilization efficiency of nutrients and resources.

## 5 Conclusion

In conclusion, we performed integrated transcriptome and metabolomics on the rumen fed with RW and RFW. RFW significantly increased levels of IgG, IgA, IgM and GLB, and induced the expression levels of immune-related pathway genes, thereby improving the immunity of sheep. Although RW could also induce some immune-related genes, its effect was far less than that of RFW. Remarkably, both RFW and RW treatment significantly increased sheep carcass weight, fat content and ash content. RW significantly affected the metabolic patterns of rumen, mainly altering the compositions of lipid acids, organic acids and organ heterocyclic compounds, thereby affecting meat quality. However, RFW only altered the compositions of organic acids and organ heterocyclic compounds in the rumen, and had little effect on the level of lipid acid. In summary, both RW and RFW could improve the production performance of sheep. RFW could further promote overall immunity in sheep. Our results illustrated the mechanism of the effect of RW and RFW on the immunity and production performance of sheep, and provided a theoretical basis and strategy for the rational utilization of *Lycium barbarum* residue feed resources.

## Data Availability Statement

The original contributions presented in the study are publicly available. This data can be found here: https://www.ncbi.nlm.nih.gov/ accession number PRJNA815909 and https://www.ebi.ac.uk/metabolights/ accession number MTBLS4507.

## Ethics Statement

The animal study was reviewed and approved by Institutional Animal Care and Use Committee of Ningxia University (NXUC20200618).

## Author Contributions

YZ and YG drafted the original manuscript and prepared the table and figure. GZ designed the experiments. YL, XY, MD, and XX provided critical feedback. All authors read and approved the submitted version.

## Funding

This study was supported by the National Natural Science Foundation of China (31960672), Top Discipline Construction Project of Pratacultural Science (NXYLXK2017A01) and the Key Research and Development Program of Ningxia Hui Autonomous Region (2021BBF02034).

## Conflict of Interest

The authors declare that the research was conducted in the absence of any commercial or financial relationships that could be construed as a potential conflict of interest.

## Publisher’s Note

All claims expressed in this article are solely those of the authors and do not necessarily represent those of their affiliated organizations, or those of the publisher, the editors and the reviewers. Any product that may be evaluated in this article, or claim that may be made by its manufacturer, is not guaranteed or endorsed by the publisher.

## Supplementary Material

Supplementary Figure 1 Pathway analysis on RW- and RFW-suppressed genes. Scatter plots showing the results of KEGG pathway enrichment analysis on downregulated genes upon RW **(A)** or RFW **(B)** treatment. The dot size and color represented the gene number and P value of each pathway.

## References

[1] Wang B Ma MP Diao QY Tu Y. Saponin-Induced Shifts in the Rumen Microbiome and Metabolome of Young Cattle. Front Microbiol (2019) 10:356. doi: 10.3389/fmicb.2019.00356 30873143 PMC6403146

[2] Martins N Petropoulos S Ferreira ICFR. Chemical Composition and Bioactive Compounds of Garlic (Allium Sativum L.) as Affected by Pre- and Post-Harvest Conditions: A Review. Food Chem (2016) 211:41–50. doi: 10.1016/j.foodchem.2016.05.029 27283605

[3] Castrica M Menchetti L Balzaretti CM Branciari R Ranucci D Cotozzolo E. Impact of Dietary Supplementation With Goji Berries (*Lycium Barbarum*) on Microbiological Quality, Physico-Chemical, and Sensory Characteristics of Rabbit Meat. Foods (2020) 9(10):1480. doi: 10.3390/foods9101480 PMC7603015 33081259

[4] Panyod S Wu WK Ho CT Lu KH Liu CT Chu YL. Diet Supplementation With Allicin Protects Against Alcoholic Fatty Liver Disease in Mice by Improving Anti-Inflammation and Antioxidative Functions. J Agric Food Chem (2016) 64(38):7104–13. doi: 10.1021/acs.jafc.6b02763 27584700

[5] Wanapat M Cherdthong A Phesatcha K Kang S. Dietary Sources and Their Effects on Animal Production and Environmental Sustainability. Anim Nutr (2015) 1(3):96–103. doi: 10.1016/j.aninu.2015.07.004 29767156 PMC5945976

[6] Lu F Zhai R Ruan S Yang X Alenyorege EA Wang Y. Enhancement of Ultrasound on the Dynamic Decolorization of Wolfberry (*Lycium Barbarum*) Polysaccharides. Lwt (2021) 145:111384. doi: 10.1016/j.lwt.2021.111384

[7] Abdallah A Zhang P Zhong Q Sun Z. Application of Traditional Chinese Herbal Medicine By-Products as Dietary Feed Supplements and Antibiotic Replacements in Animal Production. Curr Drug Metab (2018) 20(1):54–64. doi: 10.2174/1389200219666180523102920 29788885

[8] Choi Y Rim JS Na Y Lee SR. Effects of Dietary Fermented Spent Coffee Ground on Nutrient Digestibility and Nitrogen Utilization in Sheep. Asian-Australasian J Anim Sci (2018) 31(3):363–8. doi: 10.5713/ajas.17.0654 PMC5838341 29103281

[9] Naseer R Hashmi AS Zulfiqar-ul-Hassan Rehman H Naveed S Masood F. Assessment of Feeding Value of Processed Rice Husk for Lohi Sheep in Growing Phase. Pak J Zool (2017) 49(5):1725–9. doi: 10.17582/journal.pjz/2017.49.5.1725.1729

[10] Su Y Chen G Cai Y Gao B Zhi X Chang F. Effects of Broussonetia Papyrifera-Fermented Feed on the Growth Performance and Muscle Quality of Hu Sheep. Can J Anim Sci (2020) 100(4):771–80. doi: 10.1139/cjas-2018-0167

[11] Haro AN Carro MD de Evan T González J. Protecting Protein Against Ruminal Degradation Could Contribute to Reduced Methane Production. J Anim Physiol Anim Nutr (Berl) (2018) 102(6):1482–7. doi: 10.1111/jpn.12973 30066437

[12] Liu C Zhang L Yang J Zhang W Wang Q Zhang J. Study on the Nutritional Value and Ruminal Degradation Characteristics of Fermented Waste Vinegar Residue by N. Sitophila. Trop Anim Health Prod (2019) 51(6):1449–54. doi: 10.1007/s11250-019-01822-4 30719611

[13] Wang Y Wang R Hao X Hu Y Guo T Zhang J. Growth Performance, Nutrient Digestibility, Immune Responses and Antioxidant Status of Lambs Supplemented With Humic Acids and Fermented Wheat Bran Polysaccharides. Anim Feed Sci Technol (2020) 269:114644. doi: 10.1016/j.anifeedsci.2020.114644

[14] Arreola-Ramírez JL Vargas DMH Manjarrez-Gutiérrez G Alquicira J Gutiérrez J Córdoba G. Modifications of Plasma 5-HT Concentrations During the Allergic Bronchoconstriction in Guinea Pigs. Exp Lung Res (2013) 39(7):269–74. doi: 10.3109/01902148.2013.805855 23848294

[15] Lechin F van der Dijs B Orozco B Jara H Rada I Lechin ME. Neuropharmacologic Treatment of Bronchial Asthma With the Antidepressant Tianeptine: A Double-Blind, Crossover Placebo-Controlled Study. Clin Pharmacol Ther (1998) 64(2):223–32. doi: 10.1016/S0009-9236(98)90156-4 9728903

[16] Berger M Gray JA Roth BL. The Expanded Biology of Serotonin. Annu Rev Med (2009) 60:355–66. doi: 10.1146/annurev.med.60.042307.110802 PMC5864293 19630576

[17] Cloutier N Allaeys I Marcoux G Machlus KR Mailhot B Zufferey A. Platelets Release Pathogenic Serotonin and Return to Circulation After Immune Complex-Mediated Sequestration. Proc Natl Acad Sci USA (2018) 115(7):1550–9. doi: 10.1073/pnas.1720553115 PMC5816207 29386381

[18] Martin JA Wang Z. Next-Generation Transcriptome Assembly. Nat Rev Genet (2011) 12(10):671–82. doi: 10.1038/nrg3068 21897427

[19] Dunn WB Broadhurst D Begley P Zelena E Francis-Mcintyre S Anderson N. Procedures for Large-Scale Metabolic Profiling of Serum and Plasma Using Gas Chromatography and Liquid Chromatography Coupled to Mass Spectrometry. Nat Protoc (2011) 6(7):1060–83. doi: 10.1038/nprot.2011.335 21720319

[20] Wang J Zhang T Shen X Liu J Zhao D Sun Y. Serum Metabolomics for Early Diagnosis of Esophageal Squamous Cell Carcinoma by UHPLC-QTOF/Ms. Metabolomics (2016) 12(7):116. doi: 10.1007/s11306-016-1050-5

[21] Smith CA Want EJ O’Maille G Abagyan R Siuzdak G. XCMS: Processing Mass Spectrometry Data for Metabolite Profiling Using Nonlinear Peak Alignment, Matching, and Identification. Anal Chem (2006) 78(3):779–87. doi: 10.1021/ac051437y 16448051

[22] Brubaker SW Bonham KS Zanoni I Kagan JC. Innate Immune Pattern Recognition: A Cell Biological Perspective. Annu Rev Immunol (2015) 33(1):257–90. doi: 10.1146/annurev-immunol-032414-112240 PMC5146691 25581309

[23] Reeves RK Li H Jost S Blass E Li H Schafer JL. Antigen-Specific NK Cell Memory in Rhesus Macaques. Nat Immunol (2015) 16(9):927–32. doi: 10.1038/ni.3227 PMC4545390 26193080

[24] Kaufmann E Sanz J Dunn JL Khan N Mendonça LE Pacis A. BCG Educates Hematopoietic Stem Cells to Generate Protective Innate Immunity Against Tuberculosis. Cell (2018) 172(1-2):176–90. doi: 10.1016/j.cell.2017.12.031 29328912

[25] Sun JC Madera S Bezman NA Beilke JN Kaplan MH Lanier LL. Proinflammatory Cytokine Signaling Required for the Generation of Natural Killer Cell Memory. J Exp Med (2012) 209(5):947–54. doi: 10.1084/jem.20111760 PMC3348098 22493516

[26] McCormick RJ. The Flexibility of the Collagen Compartment of Muscle. Meat Sci (1994) 36(1-2):79–91. doi: 10.1016/0309-1740(94)90035-3 22061454

[27] Smith SB Johnson BJ. 0794 Marbling: Management of Cattle to Maximize the Deposition of Intramuscular Adipose Tissue. J Anim Sci (2016) 94(suppl_5):382–2. doi: 10.2527/jam2016-0794

[28] Moutiez M Belin P Gondry M. Aminoacyl-tRNA-Utilizing Enzymes in Natural Product Biosynthesis. Chem Rev (2017) 117(8):5578–618. doi: 10.1021/acs.chemrev.6b00523 28060488

[29] Mun CH Kim JO Ahn SS Yoon T Kim SJ Ko E. Atializumab, a Humanized anti-aminoacyl-tRNA Synthetase-Interacting Multifunctional Protein-1 (AIMP1) Antibody Significantly Improves Nephritis in (NZB/NZW) F1 Mice. Biomaterials (2019) 220:119408. doi: 10.1016/j.biomaterials.2019.119408 31394431

[30] Li L Boniecki MT Jaffe JD Imai BS Yau PM Luthey-Schulten ZA. Naturally Occurring Aminoacyl-tRNA Synthetases Editing-Domain Mutations That Cause Mistranslation in Mycoplasma Parasites. Proc Natl Acad Sci USA (2011) 108(23):9378–83. doi: 10.1073/pnas.1016460108 PMC3111296 21606343

[31] Planchais C Mouquet H. Easy Pan-Detection of Human IgA Immunoglobulins. J Immunol Methods (2020) 484:112833. doi: 10.1016/j.jim.2020.112833 32771390

[32] Gan L Zhang SH Liu Q Xu HB. A Polysaccharide-Protein Complex From *Lycium Barbarum* Upregulates Cytokine Expression in Human Peripheral Blood Mononuclear Cells. Eur J Pharmacol (2003) 471(3):217–22. doi: 10.1016/S0014-2999(03)01827-2 12826241

[33] Turner MD Nedjai B Hurst T Pennington DJ. Cytokines and Chemokines: At the Crossroads of Cell Signalling and Inflammatory Disease. Biochim Biophys Acta - Mol Cell Res (2014) 1843(11):2563–82. doi: 10.1016/j.bbamcr.2014.05.014 24892271

[34] Bhatia SK Jagtap SS Bedekar AA Bhatia RK Rajendran K Pugazhendhi A. Renewable Biohydrogen Production From Lignocellulosic Biomass Using Fermentation and Integration of Systems With Other Energy Generation Technologies. Sci Total Environ (2021) 765:144429. doi: 10.1016/j.scitotenv.2020.144429 33385808

[35] Lio J Wang T. Solid-State Fermentation of Soybean and Corn Processing Coproducts for Potential Feed Improvement. J Agric Food Chem (2012) 60(31):7702–9. doi: 10.1021/jf301674u 22799754

[36] Wang C Lin C Su W Zhang Y Wang F Wang Y. Effects of Supplementing Sow Diets With Fermented Corn and Soybean Meal Mixed Feed During Lactation on the Performance of Sows and Progeny. J Anim Sci (2018) 96(1):206–14. doi: 10.1093/jas/skx019 PMC6140954 29378011

[37] Jami E Israel A Kotser A Mizrahi I. Exploring the Bovine Rumen Bacterial Community From Birth to Adulthood. ISME J (2013) 7(6):1069–79. doi: 10.1038/ismej.2013.2 PMC3660679 23426008

[38] Nur Atikah I Alimon AR Yaakub H Abdullah N Jahromi MF Ivan M. Profiling of Rumen Fermentation, Microbial Population and Digestibility in Goats Fed With Dietary Oils Containing Different Fatty Acids. BMC Vet Res (2018) 14(1):1–9. doi: 10.1186/s12917-018-1672-0 30558590 PMC6297943

