# Supplementary figures and images for: Integrated Metabolomics and Transcriptome Revealed the Effect of Fermented Lycium barbarum Residue Promoting Ovis aries Immunity
Source: Front Immunol. 2022 Apr 8;13:889436. doi: 10.3389/fimmu.2022.889436 (PMC9024334; doi:10.3389/fimmu.2022.889436)

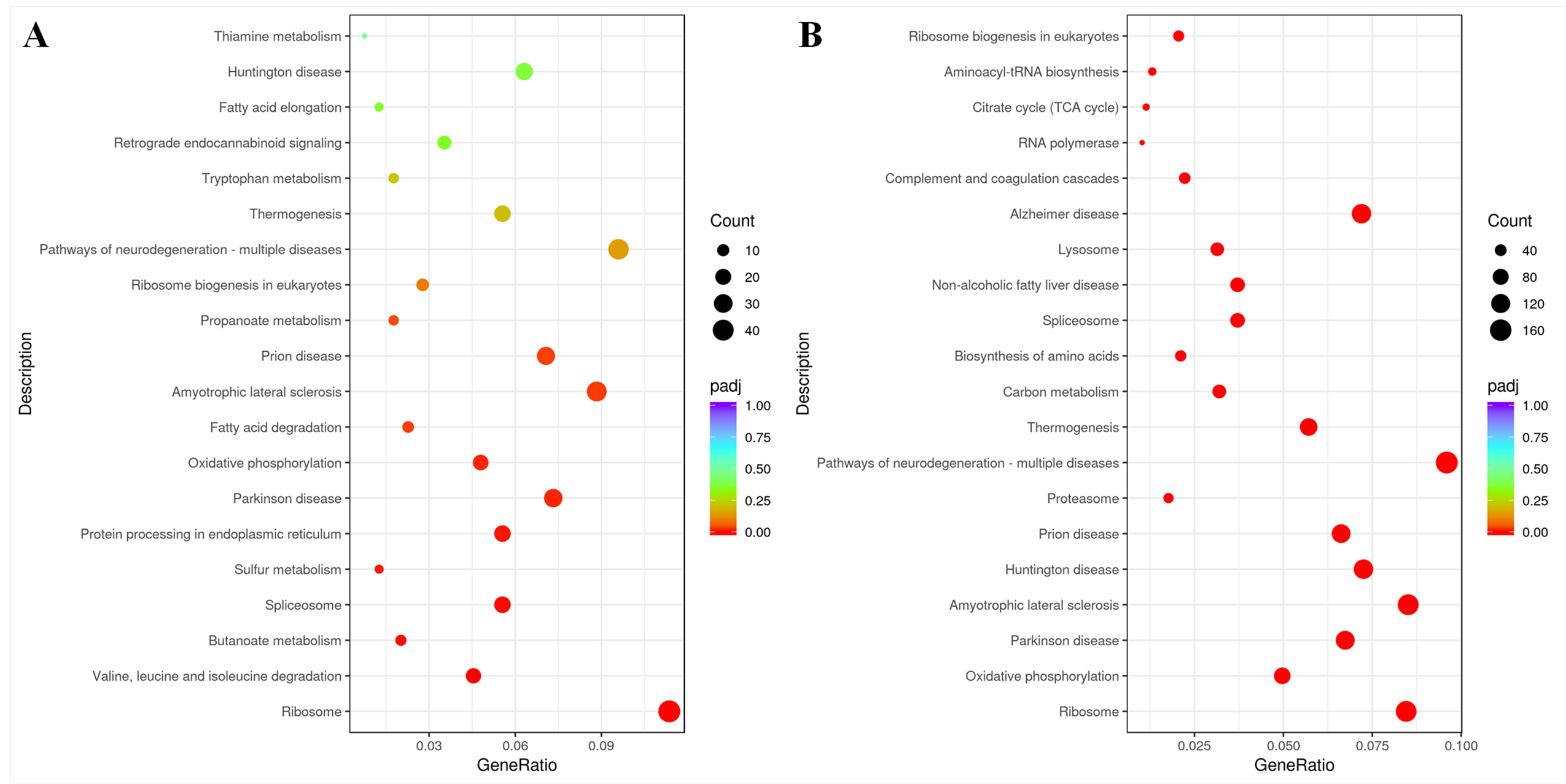

Supplement: Supplementary Figure 1 — Pathway analysis on RW- and RFW-suppressed genes. Scatter plots showing the results of KEGG pathway enrichment analysis on downregulated genes upon RW (A) or RFW (B) treatment. The dot size and color represented the gene number and P value of each pathway. [file Image_1.tif]
